# Investigation of Risk Factors, Stage and Outcome in Patients with Gestational Trophoblastic Disease since 2001 to 2011 in Iran-Yazd

**Published:** 2015-12

**Authors:** Mojgan Karimi-Zarchi, Mohammad Reza Mortazavizadeh, Malihe Soltani-Gerdefaramrzi, Mitra Rouhi, Pouria Yazdian-Anari, Mohammad Hosain Ahmadiyeh

**Affiliations:** 1 Associated professor, Department of Gynecology Oncology, Clinical Research Development, Shahid Sadoughi University of Medical Science, Yazd, Iran;; 2 Assistant professor, Hematology and oncology, hematology and oncology department, Islamic Azad University, Yazd branch, Iran;; 3 Medical Student, Student Research Committee, Shahid Sadoughi University of Medical Sciences, Yazd, Iran;; 4 General physician;; 5 Student of medicine, Scientific society of medicine, Yazd branch, Islamic Azad University, Yazd, Iran; 6 Epidemiologist, Shahid Sadoughi University of Medical Science, Yazd, Iran

**Keywords:** Gestational Trophoblastic Disease, Stage of Disease, Outcome, Risk Factors

## Abstract

**Background and Aim:**

Gestational trophoblastic disease (GTN) is one of the high-risk forms of pregnancy that requires a lot of attention in terms of research studies, considering its incidence and the importance of the disease in advanced form. The aim of this study was to investigate the risk factors and clinical procedure of patients with gestational trophoblastic disease from 2001 to 2002.

**Method of Study:**

This is a retrospective descriptive study, which was carried out on 150 patients with trophoblastic disease. These patients’ files were obtained from Shohadaye Kargar and Shahid Sadoughi hospitals and women’s oncology offices of Yazd city. The patients were contacted one by one and their disease situation was determined. The data obtained were recorded in a questionnaire and analyzed by SPSS software.

**Result:**

The results indicated that the average age of the patients was 27.65 ± 8.22 with variations in age ranging from 15 to 35 year. In addition, majorities were in the age group of 20 to 40 years. 43.2 percent of the women were affected during their first gestation. 4% had molar gestation record, and 9.4% had positive family record. Mean time of survival was 93.38 ± 0.62 months (MIN ± SE), and only one died owing to chemotherapy complication. Vaginal bleeding (90%) was the most common symptom. 54.6 percent of the disease had complete mole, 30% had incomplete mole, 8.6% had invasive mole, 4.6% had choriocarcinoma and 2% had placenta site trophoblastic tumor (PSTT). Among the patients studied, 28.7% were benign in GTN group while 71.3 % were malignant in the GTN group. The malignant patients were divided into three groups per risk, and 41.2% were in the high-risk group. There was theca-lutein cyst in 54% of the patients, which had a significant relationship with the disease risk of persistent GTN.

**Conclusion:**

Choriocarcinoma and invasive mole is the most malignant pathology. There was significant relationship between disease interval and the beginning of chemotherapy, and theca lutein cyst and persistent GTN.

## INTRODUCTION

Gestational trophoblastic disease includes a broad spectrum of benign and malignant tumors that originate from human placenta trophoblast and are divided into several groups; complete and imcomplete hydatidiform mole, invasive mole, choriocarcinoma and placenta site trophoblastic tumor (PSTT). Although their appearance is rare, but they can immediately metamorphose into a fatal disease, which can young females in gestational ages (1). Despite the obvious different types of GTN, these diseases are all derived from human placenta trophoblast, with the interference of paternal and maternal genome, which occurs randomly. Human Chorionic Gonadotrophin (HCG) is produced by this neoplasm and acts as sensitive tumor marker that is in consonance with clinical outcome of all GTN types except PSTT (2).

Appearance rate of GTN differs in various regions of the world. Approximately 3000 cases are diagnosed with hydatidiform mole and 500-750 cases are diagnosed with malignant GTN in the United States every year. In United States, the incidence of hydatidiform mole is about 1 in every 1500 gestations. In the Far East and Eastern South of Asia, this value is 5-15 times more than that in industrial Western countries. For example, mole gestation is two cases in every 1000 gestations, and this value is 3 times more than that in Europe and North America (3, 4). Risk factors known to appear in hydatidiform mole consist of previous molar gestation and mother’s age. The women with hydatidiform mole are 4-5 times at risk of molar gestation in the future (5, 6).

Vaginal bleeding is the most common symptom that compels the patient to visit a physician. Previously, the incidence of this symptom was stated to be 97%, this number has dropped to 84% (7, 8).

Respiratory distress is a common symptom in patients with oversized uterus as well as a significant increase in HCG serum level (4). Massive theca-lutein ovarian cysts (with diameter of 6 cm) appeared in the patients with complete mole (9).

Taking into cognizance the fact that trophoblastic disease are prevalent in races of Eastern Asian region, and that they have the most reports on this disease compared to Europe and American regions. Yazd Province as a referral region has accepted many of the above mentioned cases. However, there are still no statistical and accurate widespread studies on cysts of this disease to encourage researchers to observe and investigate risk factors, disease mechanism and patients’ fate completely and comprehensively within a specified duration. Our aim was to investigate the risk factors and patients’ fate as well as the general duration and the duration without disease and its complications.

## METHOD OF STUDY

This study was carried out by descriptive analytical method between 2001 and 2011 on 150 patients diagnosed with gestational trophoblastic disease. The patients’ files were obtained from the archives of Shahid Sadoughi, Shohaday Kargar, Afshar hospitals. Necessary information such as age, parity, previous molar gestation record and family background, smoking, oral contraceptive pills (OCP), blood group and pathologic diagnosis, disease symptom, age of gestation, titrage of BHCG, time of diagnosis, type of therapy, etc were obtained from the oncology department and women’s offices. The patients were called one after another and their health and medical complications were recorded. In addition, some patients who did not have files were asked questions and the information given were recorded in the previously prepared questionnaire and the data were analyzed statistically. All patients whose pathologic diagnosis fell into one of the types of GTN were included in the study and the criteria for exclusion from the study were insufficient information in patient’s file and inaccessibility of patients for diagnosis during the duration time.

## RESULTS

In this research, 150 patients with gestational trophoblastic disease whose pathologic diagnosis was confirmed were investigated between 2001 and 2011. The mean age of the studied samples were 27.65 ± 8.22 years with variation in age ranging from 15-53 years.

The average marriage age of the studied cases were 20.8 ± 3.9 years with variation ranging from 12 to 37 years. In addition, their husbands’ average age was 23.5 during the diagnosis of their disease with variation ranging from 19 to 65 years. 89 of these patients (65.5%) were resident in Yazd, 23 patients (14.9%) were resident in Sothern provinces, and 29 patients (19.6%) were resident in other provinces of Iran. The mean of the studied patients’ duration was 93.38 ± 0.62 (mean ± SD) months after diagnosis and only one patient in the studied samples was died. Thus, it was not possible to use K.M diagram drawings and Long Rank test to compare the factors affecting duration rate. Among the studied population, 65 people (43.2%) had blood group O, 29 people (19.5%) had blood group A, 45 people (30.9%) had blood group B, 11 people (7.4%) had blood group AB. In addition, among these numbers, 133 people (89.9%) were + Rh and 15 people (10.1%) were –Rh. Mean gestation age in the studied women was 10.85 ± 3.8 weeks with variations range ranging from 4 to 28 weeks. The average gestation number of the studied people was 2.3 with variations ranging from 1 to 12 gestations. Among these numbers, 70 people had been affected by GTN during their first gestation and 4 people after abortion, one person after term gestation and one person after EP had been affected by GTN. In addition, there was one case that had hydatidiform mole with fetus in two gestations among the studied samples. This phenomenon is very rare and its prevalence is 1 in every 10000-22000 gestations. The studied patients were divided into two groups of GTN and malignant GTN. 43 people (28.7%) were in GTN group and these people were cured only by curtege and their BHCG titrage decreased. They recovered and therefore had no need for chemotherapy. 107 (71.3%) people were in the malignant GTN group and these patients had chemical therapy. In addition, 68 people (63.5%) were in the metastatic group and 39 people (36.5%) were divided into non-metastatic group. Among them, 51 people (75%) had uterus metastasis (Stage I) , 8 people (11.7%) vaginal metastasis (Stage II) , 9 people (13.3%) lung metastasis (Stage III) were among the 107 people who received chemotherapy. In addition, 20 people (18.7%) received compound chemical therapy and 87 people (81.3%) received single medicine chemotherapy and from these numbers 75 people (69.4%) had proper response to chemotherapy while 33 people (30.6%) had no good response to chemotherapy due to the persistence of their disease. As was mentioned, among the studied 150 patients just one case died because of GTN disease. This 53 years old patient in her fifth gestation had been affected by the choriocarcinoma type of GTN, which metastasized to her uterus. The patient referred had symptoms of bleeding, anemia, hyperthyroid and intense nausea and vomiting. Her disease was diagnosed in the 14th week of gestation. The patient came to emergency unit because of intense hysterectomy bleeding. The patient received single medicine chemotherapy and she died because of infections and sepsis.

As seen in Table 1, the average gestation age of the studied cases was 10.89 weeks and this indicates that the disease was diagnosed in the mentioned cases at the right time.

**Table 1:** Mean of GTN Disease Risk Factors in the Studied Samples

	Mean	Stdeviation	min	Max

Patient’s age (year)	27.61	8.22	15	53
Husband’s age (year)	32.5	8.63	19	65
Age of gestation (week)	10.89	3.92	4	28
BHCGTitrage	227984.79	883724	102	8782082
BMI	23.8	4.28	14.69	36.68

As seen in Table 2, about 6% of the patients with GTN disease had a history of this disease in their previous gestation. Considering GTN statistics in the normal population that has been reported, 1.8 in every 1000 gestation (6) shows that molar gestation record can be attributed as an important risk factor in a person. In addition, as seen, 9.4% of the studied patients had positive family history and considering statistics, this history can be attributed as a GTN disease risk factor.

**Table 2:** Abundance distribution of risk factors in the under studied GTN samples

	Total		Yes		No	
	Number	%	Number	%	Number	%

Molar gestation Background	141	94	9	6	150	100
GTN family Background	136	90.6	14	9.4	150	100
Consumption of Ocp	108	72	42	27.3	150	100
Presence of theca-lutein cyst	81	54	54	36	135	90

The majority of the studied people were not smokers (60.7%) and only 4 people were active smokers (2.7%) while 54 people (36%) were passive smokers.

As observed in Table 3, the most common symptom appears during diagnosis of bleeding in the disease (90%) and this is why about 15 people (10%) in the studied sample did not record symptoms of bleeding.

**Table 3:** Abundance distribution of disease symptoms during diagnosis time in the under studied samples

Symptoms	No		Yes	
	t	d	t	d

Bleeding	15	10%	135	90%
Intense nausea and vomiting	100	67%	50	33%
Overall sized uterus	117	78%	33	22%
High blood pressure	145	96%	5	4%
Sonography person	50	33%	100	67%
Anemia	138	92%	12	8%
Hyper thyroid	142	95%	8	5%

12 cases (8%) of these people were affected by anemia due to intense bleeding. Hyperthyroid, which is a symptom of the disease, appeared because of the high BHCG level in 8 people (5%) of the studied group.

The most common pathology among the studied patients was complete mole (54.6%) and PSTT had less value (2.2%). Other types of pathology were incomplete mole (30%), invasive mole (8.6%) and choriocarcinoma (4.6%).

Abundant distribution of blood group per disease risk factor (high, mid and low) was done and the data were analyzed via Chi-square test, and there was no relationship between blood group and disease risk factor (*P* value=0.49).

## DISCUSSION

This study showed there was significant relationship between disease diagnosis interval, and the beginning of chemotherapy and disease risk. In the study carried out between 1994 and 1997, epidemiology of trophoblastic disease was investigated in Tehran Shariati hospital. 48 patients were investigated in this study and their age mean was 20-25 years. 30 people (62%) had mole gestation and 18 people (37.5%) had gestational trophoblastic tumor. 93 percent of the patients had dangerous gestation. They concluded that many (93%) of the studied patients had risk factors, and it seems that their resistance against the disease was higher (13).

In the study carried out between 1998 and 2007, an abundant level of hydatidiform mole and its relevant risk factors were investigated. This study was carried out by descriptive analytical method on 200 hydatidiform patients as a case study group and 400 women who were referred for childbirth as evidence group in Kermanshah hospital. In this study, abundance level of hydatidiform mole was 3.1 in 1000 live births and abundance level of complete and incomplete mole was 2.07 in 1000 and 1.04 in 1000 live births respectively. Average age of patients with hydatidiform mole was 26.33 years, and 60% of the patients were multipar. Among the studied factors, the presence of molar gestation history and irregular menestural periods had a significant effect on the disease abundance (14). In the study carried out by Usefi *et al*., the abundance of resistant mole in patients with GTN was investigated. This study was carried out between 1991 and 2004 on 250 patients who were referred to Qaem hospital. Among these number, 74 patients had resistant mole and their average age was 25.6 years. 44.6% of the patients had O positive blood group, 43.9% of them had 9-12 week gestation and 100% of the studied people recovered completely (15). In a study carried out by Mungan *et al*., between 1989 and 1994 in Turkey, prevalence of molar gestation was estimated to be 1.84 in every 1000 gestations. Average age of the studied patients was 25.29 years and 60% of the patients were affected by this disease in their first gestation. 5.5% of the studied cases had molar gestation history and 8 patients had two histories.

The most common symptom among the patients (71%) was vaginal bleeding, 17% of the people have had theca-lutein cyst. 14.5% of the patients were refractory to treatment. They concluded that the previous age records of illness, size of the uterus, theca-lutein cyst and BHCG titrage cannot be used as a proper criteria to determine prognosis in refractory patients by comparing spontaneously improved patients with persistent patients (11, 16). In Basirat *et al*., a study which was conducted in Zabol on 40 hydatidiform mole patients between 1997 and 2001, the most common symptoms reported at the time of diagnosis were vaginal bleeding (95%), anemia (15%), enlargement of uterus (50%), severe hyperemesis (25%), hyperthyroidism (2.5%) and there were no cases of preeclampsia and pulmonary embolism. They concluded that noticing clinical symptoms and ultrasound early in pregnancy leads to early diagnosis and reduced complications as well as decreased stability of the disease (17). In a study conducted [1994-2003] in Sanandaj Besat hospital, the GTN prevalence was estimated to be 2.02 per 1000 pregnancies. They examined 81 patients. 78 cases had hydatidiform mole (96.3%), two of them (2.5%) had invasive mole and one (1.2%) had choriocarcinoma. The mean age was 27.2 years. Given that that the prevalence of the disease in Sanandaj was similar to that in other regions in Iran, they suggested however, that routine ultrasound must be done in the first quarter of the year in order to carry out an early diagnosis of the disease (11). In a study done in Syria between 2007 and 2009, the relationship between maternal age and GTN disease was examined. Sixty (60) patients were studied and the prevalence of partial mole and complete mole was 60% and 40% respectively. The prevalence of complete mole in patients < 20 years old was higher than the prevalence of partial mole in patients < 35 years old. It was concluded in this study that maternal age is a risk factor for molar pregnancy (16). In the study carried out [2011] in Pakistan, 1056 pregnant women who had gone to Nawabshah hospital were studied. 30 of them suffered from GTN. Its prevalence was estimated to be 28 per 1000 live births. 21 women (70%) had hydatid mole, 7 had (23.3%) invasive mole and 2 patients (6.6%) had choriocarcinoma. 23 (76.6%), 25 (83.3%) and 4 (13.3%) women were treated with chemotherapy, suction curettage and hysterectomy respectively. 29 patients recovered fully while one died due to brain metastases. They concluded that its prevalence is higher than what international studies have reported and that hydatid mole is the most common form of the disease while the most common symptom is vaginal bleeding and pelvic pain (18). This study reviewed the records of 150 patients with GTN and it was suggested that the mean age of patients with this disease was 27.65 years, which is consistent with the results of the research carried out by Farhadi *et al*., and studies conducted in France (16). According to the report that 65.5% of the studied patients live in Yazd, and Yazd is a medical referral center for other regions in Iran, prevalence of the disease cannot be accurately estimated, but based on statistical studies, prevalence of the disease in Yazd is 1.8 per 1000 pregnancies (11). The overall survival rate of the studied patients from the time of diagnosis to the period of follow-up was 93.38 months, and only one patient was observed to have died due to complications of chemotherapy. Most of the people in the study were 20 to 40 years old. In the study, the incidence of mole was higher among 15 years old women and those younger as well as women older than 45 years. However, it should be noted that the high number of people with mole in the age range of 20 to 40 years could be affected because they experience more pregnancies than older women do. The mean age of the studied persons’ spouse at the time of diagnosis was equal to 23.5, while a case control study found that women whose partners are over 40 years old are most prone to the disease (10). Numbers of pregnancies as a risk factor for hydatid mole is a variable, which has not been fully known yet. The mean number of pregnancies in our study was 2.3. In this study, 70 patients (46.6%) were experiencing their first pregnancy and the mean gestational age was 10.89 weeks while in 6% of the studied persons, subjects had a history of molar pregnancy (12). In addition, it was found in Lorigan study that people with a history of molar pregnancy are at a higher risk of significant recurrence than the general population. The reason seems to be unclear but previous molar pregnancy could be a factor. In our study, 9.4% of patients had a positive family history, which was not noted in any other studies. In this study, 43 % had blood group O, which is in consonance with the study of Ziaei *et al*., but in the research carried out by the Basirat *et al*., blood group B was a risk factor for suffering from molar pregnancy (18). In the study, blood group A was identified as a risk factor. 89.9% of the studied subjects had positive Rh, which was consistent with Ziaei’s results (18). In the study, the relationship between Rh and the disease was not significant (9). The differences may be due to the different distribution of blood groups in several regions, genetic and environmental differences. Due to the different results obtained, the relationship between the incidence of mole and blood group has remained unknown.

60% of the studied population had no mentioned history of active or passive smoking, and 72% of them had no history of using the OCP. 74.8% had a BMI below 27. Accordingly, high smoking and OCP as well as BMI cannot be regarded as a risk factor for the disease while these are mentioned as a risk factor in textbooks (20, 21). Vaginal bleeding was the most common symptom of patients in this study (90%), which was consistent with most studies while the most common symptoms was identified as enlargement of the uterus in Ziaei *et al*. Anemia and hyperthyroidism were observed in 8% and 5% of the patients respectively while Felemban *et al*., in Saudi Arabia mentioned 15.5% of patients to have had severe anemia while references had mentioned anemia to be 50% (1). Perhaps, the reduced prevalence of anemia in our patients can be associated with more rapid disease detection due to the increase in the use of ultrasound during pregnancy related care. 54% of the patients had theca-lutein cyst while Basirat *et al*. (17), observed no theca-lutein cyst in his study. Reliable references predict the incidence of the cyst to be 50% (1). Hydatidiform moles may develop into choriocarcinoma. From the viewpoint of trophoblastic disease, the results of our study were 54.6% complete mole, 30% partial mole, 8.6% invasive mole, 4.6 % choriocarcinoma and 2% PSTT, which was consistent with the French study but differed significantly from studies carried out in South Africa (70% mole and 30% choriocarcinoma). The reason for this difference is unclear. It could be due to race or more likely due to problems and weakness in pathology. In this study, patients were divided into two groups of GTD (Gestational Trophoblastic Disease) (28.7%) and malignant GTN (71.3%). The first group received no chemotherapy while the second group was treated with chemotherapy. In a study carried out on 48 patients, 62.5% had benign GTN or GTD while 37.5% had malignant GTN. In this study, patients were divided into two groups of low risk and high risk according to the presence of existing enlargement in the uterus and BHCG titrage < 4000 and theca-lutein cyst. Of 93.5% of patients with high risk GTN, there was large theca lutein cyst and high titrage of BHCG. However, the malignant patients in our study were classified into three groups of patients with high risk (41.2%), middle risk (50.4%) and low risk (8.4%). Such division was not done in any of the studies and the high-risk levels obtained were consistent with reference books (41%). There was a significant interaction between pathological type of the disease and risk of persistant disease with *P* value = 0.005 in our study, which was also mentioned in reliable references that choriocarcinoma and invasive mole is the most malignant pathology (20) and was in high risk groups in the study. In the study, there was a significant relationship between theca-lutein cyst and the risk *P* value = 0.036 which was also noted in reliable references (5). Among the studied patients in the malignant group, 68 patients had metastasis and 39 patients were without metastasis of which 51 (75%) patients had disease confined to the uterus. The findings were obtained after the examination of metastasis (chest radiography, ultrasound of the abdomen and pelvis, brain scans). In this way, 75% of metastatic disease were in stage I, and 8 patients experienced metastasis of the vagina or pelvis (stage II) and 9 (31.3%) had metastatic lung (stage III). 20 malignant patients (18.7%) and 87 patients (81.3%) were treated with combination chemotherapy and single agent chemotherapy respectively. At present, the combination regimen of EMA / CO was introduced as initial therapy in patients with metastatic and high-risk GTN (7) and 56.8% of those in high risk groups did not respond to initial chemotherapy. It was also found that there was a relationship between the time interval from diagnosis to initiation of chemotherapy and the risk of disease with *p* value = 0.02. The longer the interval time, the higher the risk of the disease.

## SUGGESTIONS

According to the results obtained, it can be claimed that the majority of the studied subjects had been in middle or high-risk groups and a longer time from diagnosis to initiation of treatment is recommended to increase the risk of the disease. So, in order to speed up early diagnosis of this illness, it is suggested that routine ultra sonography be carried out for patients in the first trimester of pregnancies and more attention should be paid to clinical findings. In addition, support centers should be provided for patients with GTN so that prospective studies can be performed with a larger population size in the centers. It shall be a big step for early diagnosis and early treatment of the disease as well as decreasing the number of cases, and by extension reducing costs and helping to improve the economy and promote our country.
